# Region Duplication Forgery Detection Technique Based on SURF and HAC

**DOI:** 10.1155/2013/267691

**Published:** 2013-11-07

**Authors:** Parul Mishra, Nishchol Mishra, Sanjeev Sharma, Ravindra Patel

**Affiliations:** ^1^School of Information Technology, RGPV, Bhopal, Madhya Pradesh 462036, India; ^2^University Institute of Technology, RGPV, Bhopal, Madhya Pradesh 462036, India

## Abstract

Region duplication forgery detection is a special type of forgery detection approach and widely used research topic under digital image forensics. In copy move forgery, a specific area is copied and then pasted into any other region of the image. Due to the availability of sophisticated image processing tools, it becomes very hard to detect forgery with naked eyes. From the forged region of an image no visual clues are often detected. For making the tampering more robust, various transformations like scaling, rotation, illumination changes, JPEG compression, noise addition, gamma correction, and blurring are applied. So there is a need for a method which performs efficiently in the presence of all such attacks. This paper presents a detection method based on speeded up robust features (SURF) and hierarchical agglomerative clustering (HAC). SURF detects the keypoints and their corresponding features. From these sets of keypoints, grouping is performed on the matched keypoints by HAC that shows copied and pasted regions.

## 1. Introduction

Today, the use of digital images is increasing rapidly in almost every area of human life like in education, software companies, television, businesses, journalism, medical imaging, and social media. It is easy to learn and understand anything visually rather than only reading or listening. Another aspect is that generally visual information is believed to be true. But as the technology advances and lots of sophisticated image processing tools are available, it becomes very easy to edit visual information. Some of the tools are *Adobe Photoshop*, *GIMP*, *Macromedia Freehand*, and *Corel Paint Shop *[1, 2]. A big question arises, how to distinguish the photographic images from the photorealistic ones [3]. 

Digital image forensic is a branch that deals with crimes, where images are used as a prime evidence in the court of law. Forensic sciences have methods to identify the source device, for example, camera, scanner, and so forth, with their particular model. If any tampering is done on the image then it can also be detected. Tampering an image means either adding or removing some information from an image, so that the original meaning will be changed [4]. 

If the alteration is intentional and related with some kind of benefits, it can be termed as a digital image forgery. Forgery detection methods are mainly divided into two different categories: *active* and *passive*. In active method, some information for example digital watermark or digital signature is preembedded into the image. This procedure is performed for the sake of providing authenticity to an image. But the images distributed on the web do not contain always preembedding information. To overcome the drawback of active methods, passive methods have been developed [5]. The tampering again is of two types either it is performed on the same image or on multiple images. If some area is copied and pasted into another area of the same image, it is known as *copy move forgery *or* region duplication forgery*. When two or more images are involved and their combination produces a fake image then it is called *splicing* or *photomontage* [6]. Farid gives lots of examples of real incidents with image forgery [7]. On July 2008, a forged image of four Iranian missiles was posted on the web and published in newspapers [8]. Egypt's newspaper *Al-Ahram* on Sept. 2010 published a forged image. In this image president Mubarak was leading the group instead of Barak Obama at the White House, during Middle East peace talks [9]. 

The rest of the paper is organized as follows: Section 2 presents the related work; Section 3 describes the proposed method for duplicate detection. The results of forgery detection by experimental evaluation are presented in Section 4, and Section 5 describes the conclusion of the paper.

## 2. Related Work

**Figure 1:**
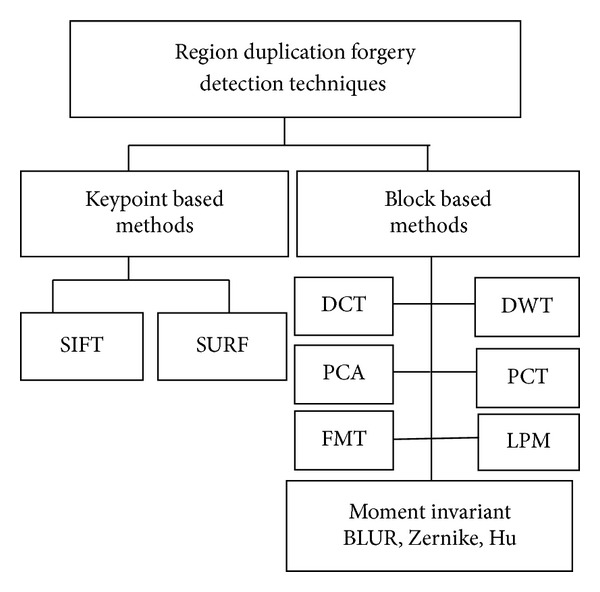
Classification of copy move forgery detection techniques.

In region duplication detection, the forged region can be identified by applying proper detection techniques. These techniques are classified into block based and keypoint based methods [10]. The classification of duplication detection methods is illustrated in Figure 1.

### 2.1. Block Based Methods

In this approach, an image is divided into different overlapping blocks of fixed size; it is assumed that the block size is smaller than the duplicated region. After dividing the image into different block sizes, features are extracted from it by applying different methods. These features are then matched with other features of each block. A match indicates the probability of forgery. Fridrich et al. [11] discuss the exhaustive search and autocorrelation of forgery detection. Furthermore, they applied discrete cosine transform (DCT) on each separate block. DCT is applied from the upper left corner to the bottom right corner. For reducing the computation, features are extracted from the low frequency component. In this method, feature dimension size is 64.

Popescu and Farid [12] presented a method based on principal component analysis (PCA) that reduces the dimensional size to 32. Extracted features are lexicographically sorted; therefore, matched features come closer to each other. This method is robust towards compression and additive noise. Bayram et al. [13] proposed Fourier Mellin transforms (FMT) for copy move forgery detection. Features extracted are of length 45, and they are rotation invariant only to some degree. Bloom filter was used here instead of lexicographical sorting, which reduces the detection time.

Moment invariant features are insensitive towards all transformation; hence, it can be used to detect the region duplication. Blur moment invariant detects the forgery in the presence of blur degradation, and its performance remains unaffected by additive zero mean noise. Each separate block is represented by a feature vector whose dimension length is 24 in case of gray level. The KD tree method is applied here for nearest neighbour searching, and then similar blocks denote the duplicated regions [14]. Ryu et al. [15] proposed a method which utilizes rotation invariant Zernike moment. It also gives significant performance in case of JPEG compression, blurring, and noise contamination. In [16], the authors applied Gaussian pyramid on image to decompose it into different scales. After that each scale space is divided into separate circular blocks. From each circular block features are extracted by Hu moment of length 4. Features are then sorted, and matching is performed on it. The performance of this method is not changed in the presence of a rotational transformation.

Li et al. [17] applied discrete wavelet transform (DWT) and singular value decomposition (SVD) to the image. Firstly, DWT is applied to each block that reduces the size of the block. Then, SVD is applied on low frequency components, which reduce the feature dimensional size to 4. After that features are lexicographically sorted and matched. In [18], the image is divided into overlapping patches. Features are extracted from each patch by applying PCT. PCT utilizes the orthogonal properties so that features of PCT are more compact. It is a rotational invariant method and also performs better in the presence of noise.

Bravo-Solorio and Nandi [19] mapped all the pixels of overlapping blocks into log polar coordinates. Then, angle orientation creates a 1D descriptor, by doing this synchronization problem can be removed. Feature vectors are calculated from particular blocks depending on the colour and luminance factor. This method is robust towards reflection, rotation, and scale changes.

### 2.2. Keypoints Based Methods

In this approach, various keypoints are selected from an image. Feature descriptors are calculated from each of these keypoints. For detecting the duplication, forgery matching is performed on keypoint feature descriptors. Lowe [20] invented SIFT, which detects keypoints and features from an image. SIFT application exists in various fields. It performs better in comparison to previous descriptors [21].

In [22], SIFT is applied into the region duplication detection method. In [23], the authors suggested that the keypoint matching method suffers from some problems. For removing these problems, SIFT cluster matching is proposed, where objects are matched rather than the point. Points are grouped here using agglomerative hierarchical clustering.

Pan and Lyu [24] also applied SIFT in their work. For avoiding search from close adjacency, search is applied far from 11 × 11 pixel window whose center is at the keypoint. They also applied Random Sample Consensus (RANSAC) for affine transformation detection.

Amerini et al. [25] detect the duplication forgery and also estimate the transformation by RANSAC. They developed g2NN nearest neighbour searching for multiple copy paste detection. Their method is robust to all transformation attacks. Their procedure also works effectively for splicing attack detection. The similar work is done in [26] where the authors used MPEG7 image signature tools for extracting the features. Least Median of Squares (LMedS) algorithm is used instead of RANSAC for estimating the geometrical transformations. Recently Amerini et al. [27] proposed J linkage for effective clustering. In [28], the authors applied resampling traces with SIFT to distinguish the original region from the pasted region in a tamper image. SIFT ring descriptors are applied to an image for detecting the tampering. The size of feature dimension is reduced to 24 from 128 that increases the speed. These feature descriptors are rotationally invariant [29]. SURF is another keypoint based method, which detects 64 feature descriptors. Obtained keypoints are divided into two subsets. Matching procedure is applied and repeated until one keypoint remains in a set [30]. In [31], SURF with KD tree method are used for the detection of a particular forged region. In this proposed work, HAC method is applied with SURF for a more accurate result in terms of all attacks.

## 3. Proposed Method

This proposed method is based on a SURF algorithm for the detection of keypoints and for extracting their corresponding feature descriptors. Matching is performed in between selected keypoints by applying best bin first search procedure.

**Figure 2:**
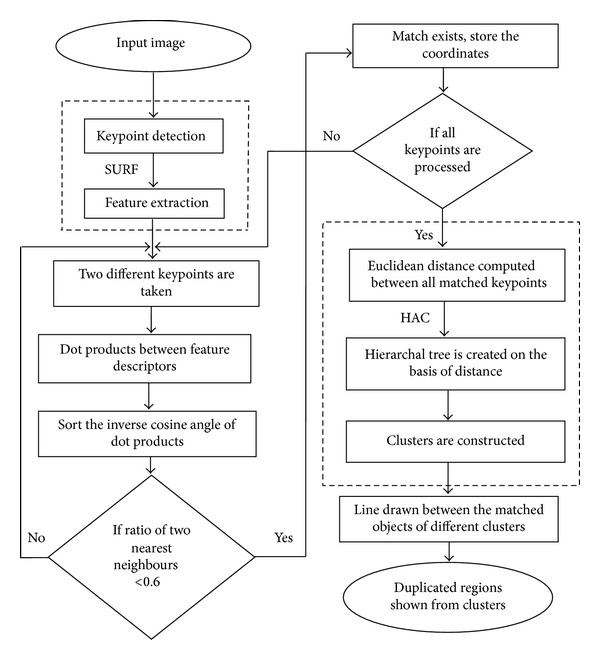
Flow chart of proposed work.

For detecting the duplicated regions, HAC technique is applied. The whole procedure of proposed work is depicted in Figure 2, and the related algorithm for detection method is described in Section 3.1. An input image is inserted to the detection system, and the output is imaged with duplicated regions, if it is forged. The first block of the detection framework is keypoint detection and feature extraction that will be explained in Section 3.2. After that matching is performed among selected keypoints, the procedure for keypoint matching will be described in Section 3.3. At last clustering algorithm is applied on the matched keypoints, which will be explained in Section 3.4. 

### 3.1. Region Duplication Detection Algorithm

If the image suffers from duplication forgery, then it contains at least two same regions, one is copied and the other is pasted region. The overall technique for detecting the duplicated region is as follows.

Input: image.

Output: detected duplicate regions with image.

(1) If RGB image, then converted into gray scale.
(2) Applying SURF method.
  (a) Keypoints are detected from an image (1,2, 3,…, *M*).
  (b) From the above detected keypoints, features are extracted (*N*_1_, *N*_2_, *N*_3_,…, *N*_*M*_).
  (c) This matrix is stored in a variable *D* = *M* × *N*.
(3) For each *i* = 1 to *M*,
 for *j* = 1 to *M*.
  (a) If *i* ≠ *j*, then go to step (b); else: return.
  (b) Dot products are calculated between each feature descriptor.
 End of For *j*.
  (c) Inverse cosine angle of dot products will be computed.
  (d) Sorting is applied on the result, and values are stored
 [Value, index] = sort (cos⁡^−1^(dot prods)).
  (e) If (Value(1)/Value(2)) < 0.6, then match exists, and index will be stored.
 Else: index = 0.
   End of For *i*.
(4) For each keypoint
  (a) If match exists, then go to step (b);
 else: return.
  (b) If the matched points are far from 10 × 10 square region, then go to step (c);
 else: return.
  (c) Store the coordinates of matched points in *m* by *n* data matrix *X* and set: flag = 1.
 End of For.
(5) If flag > 0, then
  (a) euclidean distance computed between each pair of objects of *X*;
    (1) $$\begin{array}{c} \text{Dist}\;\left(A,B\right)=\sqrt{{\left(x_{2}-x_{1}\right)}^{2}+{\left(y_{2}-y_{1}\right)}^{2}}; \end{array}$$
  where, *A* = (*x*_1_, *y*_1_  ), *B* = (*x*_2_, *y*_2_)
  (b) linkage function is applied for linking the objects into hierarchal tree;
  (c) the smallest height is taken for cutting the hierarchal tree into clusters;
  (d) a line is drawn between the matched objects from different clusters;
  (e) objects of different clusters are shown from the different colours.

### 3.2. Keypoint Detection and Feature Extraction

Bay et al. [32] proposed SURF method whose computation is faster than SIFT. How keypoints are detected and feature descriptor is generated from SURF are discussed below.

#### 3.2.1. Integral Image

Integral image increases the computation speed as well as the performance, its value is calculated from an upright rectangular area. 

**Figure 3:**
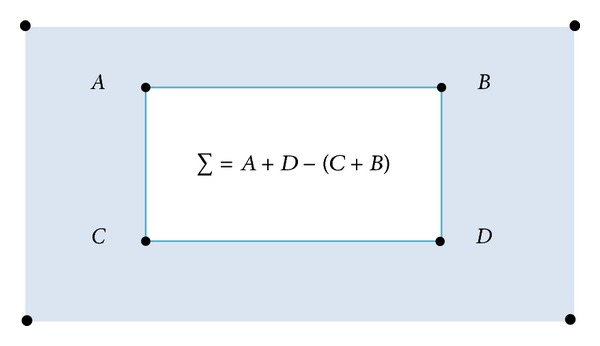
Integral image calculation by rectangular region.

In Figure 3, the sum of all pixel intensities is calculated by the formula, which is written in the rectangular area whose vertices are *A*, *B*, *C*, and *D*. Suppose that an input image *I* and a point (*x*; *y*) are given. The integral image *I*_Σ_ is calculated by the sum of the values between the point and the origin. The following formula is used to calculate the integral image:

(2) IΣ(x,y)=∑i=0i≤x∑j=0j≤y  I(x,y).

#### 3.2.2. Keypoint Detection

This step requires scale space generation for the extraction of keypoints. In SURF, Laplacian of Gaussian is approximated with a box filter. Convolution is applied to an image with varying size box filter for creating the scale space. After constructing the scale space, determinant of the Hessian matrix is calculated for detecting the extremum point. If determinant of the Hessian matrix is positive, that means both the Eigen values are of the same sign either both are negative or both are positive. In case of the positive response, points will be taken as extrema; otherwise, it will be discarded.

Hessian matrix is represented by

(3) $$\begin{array}{c} H\left(x,\sigma \right)=\left[\begin{array}{cc} L_{xx}\left(x,\sigma \right) & L_{xy}\left(x,\sigma \right)\\ L_{xy}\left(x,\sigma \right) & L_{yy}\left(x,\sigma \right) \end{array}\right], \end{array}$$

where *L*_*xx*_(*x*, *σ*) is the convolution of the Gaussian second order derivative with the image *I* in point *x*, and similarly *L*_*xy*_(*x*, *σ*) and *L*_*yy*_(*x*, *σ*). These derivatives are called Laplacian of Gaussian. The approximate determinant of the Hessian matrix is calculated by

(4) $$\begin{array}{c} \det\left(H_{\text{approx}}\right)=D_{xx}D_{yy}-{\left(0.9D_{xy}\right)}^{2}. \end{array}$$

#### 3.2.3. Orientation Assignment

At first, a circular area is constructed around the keypoints. Then, Haar wavelets are used for the orientation assignment. It also increases the robustness and decreases the computational cost. Haar wavelets are filters that detect the gradients in *x* and *y* directions. In order to make rotation invariant, a reproducible orientation for the interest point is identified. A circle segment of 60° is rotated around the interest point. The maximum value is chosen as a dominant orientation for that particular point. 

#### 3.2.4. Feature Descriptor Generation

For generating the descriptors, first construct a square region around an interest point, where interest point is taken as the center point. This square area is again divided into 4 × 4 smaller subareas. For each of these cells, Haar wavelet responses are calculated. Here, *d*_*x*_ termed as horizontal response and *d*_*y*_ as vertical response. For each of these subregions, 4 responses are collected as

(5) $$\begin{array}{c} v_{\text{subregion}\;}=\left[\sum dx,\sum dy,\sum \left|dx\right|,\sum \left|dy\right|\right]. \end{array}$$

So each subregion contributes 4 values. Therefore, the descriptor is calculated as 4 × 4 × 4 = 64.

### 3.3. Keypoint Matching

A set of keypoints and their corresponding feature descriptors are obtained from SURF. The comparison is performed between each keypoint with the remaining other keypoints feature descriptor. As matching these keypoints with their high dimensional feature vector 64 takes time, therefore best bin first (BBF) method is chosen for selecting two nearest neighbours [33]. Dot products are calculated between each keypoint feature descriptor with the others. After that sort the inverse cosine angles of dot products. Store their values as well as their corresponding index number. The ratio between two nearest neighbours value is compared to a predefined threshold. In this work, the threshold is set to 0.6, because above this value the probability of false matches arises. If the ratio is less than the given threshold, they satisfy the similarity criterion and match exists. In case of matching, their relative index number will be stored. This procedure continues for all keypoints. 

### 3.4. Keypoint Clustering

HAC is also known as hierarchy of clusters, in which each keypoint behaves as a single cluster at the starting stage. Euclidean distance between each keypoint with the remaining other keypoints will be calculated. Merging is performed if two clusters are dissimilar to each other. This step is repeated until there is one cluster left or dissimilarity criterion unsatisfied [34]. Single, average, and ward methods are types of linkage used for merging and creating a hierarchal tree.

*Single Linkage.* It uses the smallest distance between objects in two clusters,

(6) $$\begin{array}{c} d\left(A,B\right)=\min\left(\mathrm{dist}\left(x_{A_{i}},x_{B_{j}}\right)\right). \end{array}$$

*Average Linkage.* It uses the average distance between all pairs of objects in the two clusters,

(7) $$\begin{array}{c} d\left(A,B\right)=\frac{1}{n_{A}n_{B}}\;\sum_{i=1}^{n_{A}}\sum_{j=1}^{n_{B}}\mathrm{dist}\left(x_{A_{i}},x_{B_{j}}\right). \end{array}$$

*Ward Linkage.* It is based on the increment or decrement in the value of error sum of squares (ESS). In other words, distance between the clusters is the difference between the ESS for unified cluster and ESS of the individual clusters,

(8) $$\begin{array}{c} d\left(A,B\right)=\text{ESS}\left(AB\right)-\left[\text{ESS}\left(A\right)+\text{ESS}\left(B\right)\right], \end{array}$$

where

(9) $$\begin{array}{c} \text{ESS}\left(A\right)=\sum_{i=1}^{n_{A}}{\left|x_{A_{i}}-{\overset{-}{x}}_{A}\right|}^{2}. \end{array}$$

Here, *AB* indicates the combined cluster, *n*_*A*_ indicates number of objects in cluster *A*, *n*_*B*_ indicates number of objects in cluster B, *x*_*A*_*i*__ indicates *i*th object in the cluster *A*, and ${\overset{-}{x}}_{A}$ indicates centroids of cluster whose value is calculated by

(10) $$\begin{array}{c} {\overset{-}{x}}_{A}=\frac{1}{n_{A}}\sum_{i=1}^{n_{A}}x_{A_{i}}. \end{array}$$

## 4. Experimental Results 

**Figure 4:**
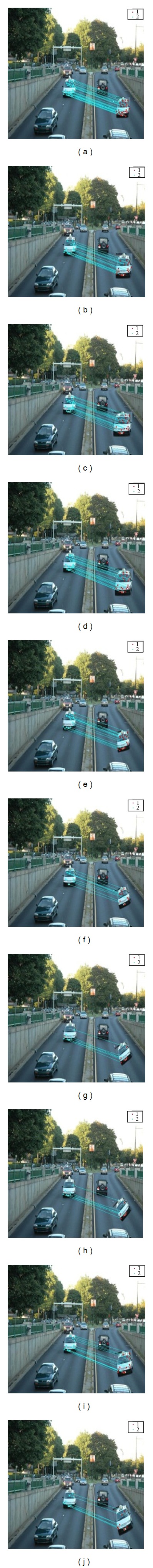
Copy paste detection results in the presence of 10 different rotation and scaling attacks applied on the image.

In this section, experiment of duplication detection is performed on the MICC-F220 dataset [35]. This dataset contains 220 images, from them 110 are real and 110 are fake. 10 different combinations of scaling and rotation attacks are already applied to each forged image of the dataset [25]. Images shown in Figure 4 represent the detection results in the presence of various scaling and rotation attacks.

**Figure 5:**
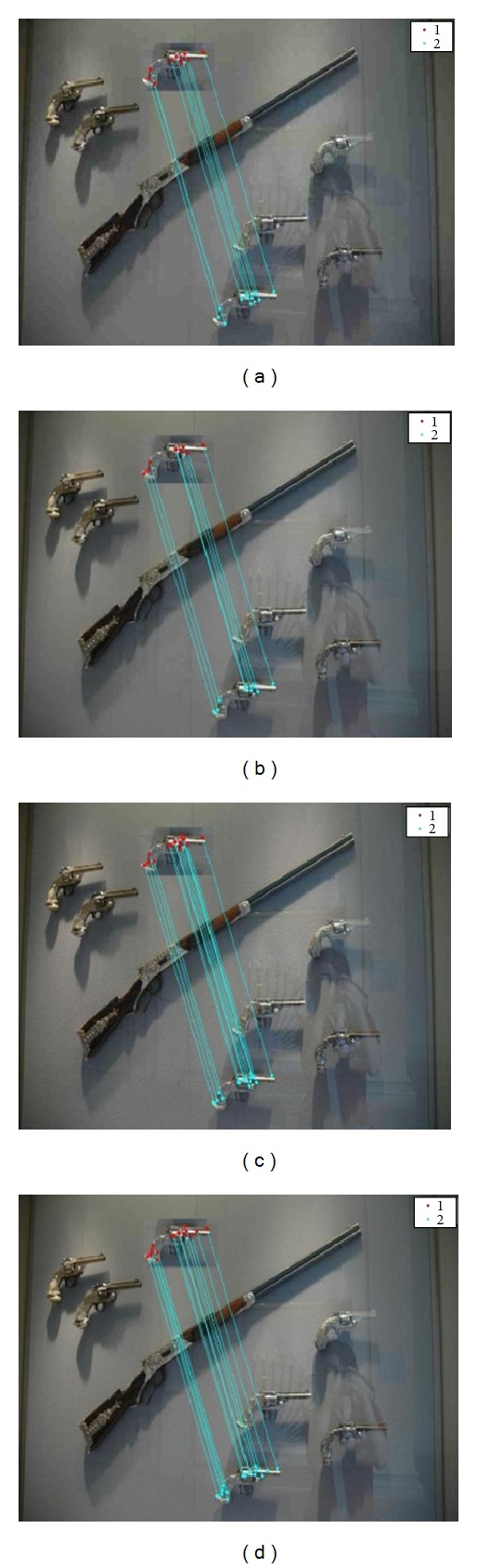
Copy paste detection results for compression: (a) JPEG image quality factor 20, (b) JPEG image quality factor 40, (c) JPEG image quality factor 60, and (d) JPEG image quality factor 80.

**Figure 6:**
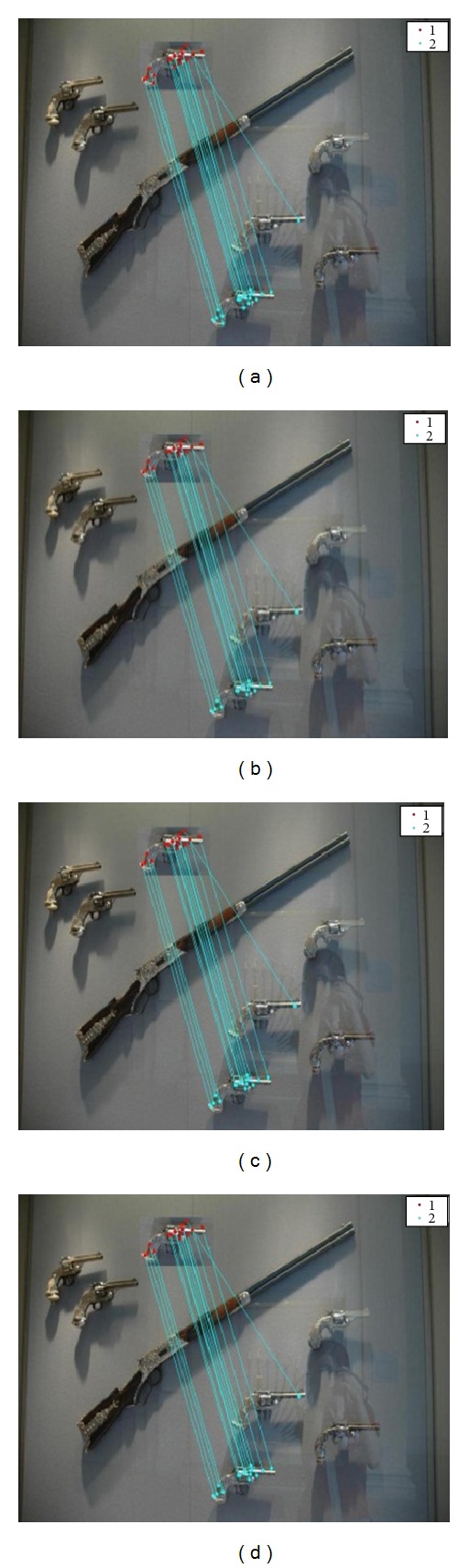
Copy paste detection results for noise addition: (a) SNR value 20, (b) SNR value 30, (c) SNR value 40, and (d) SNR value 50.

**Figure 7:**
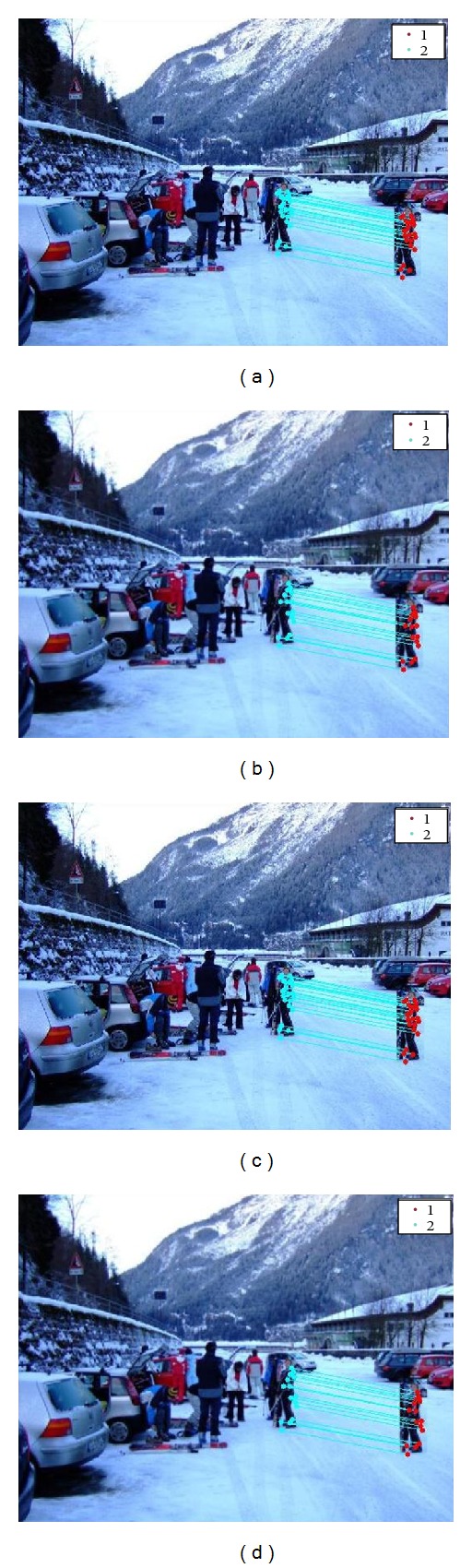
Copy paste detection results for blurring: (a) window size 5 × 5, *σ* = 0.5, (b) window size 5 × 5, *σ* = 1, (c) window size 7 × 7, *σ* = 0.5, and (d) window size 7 × 7, *σ* = 1.

**Figure 8:**
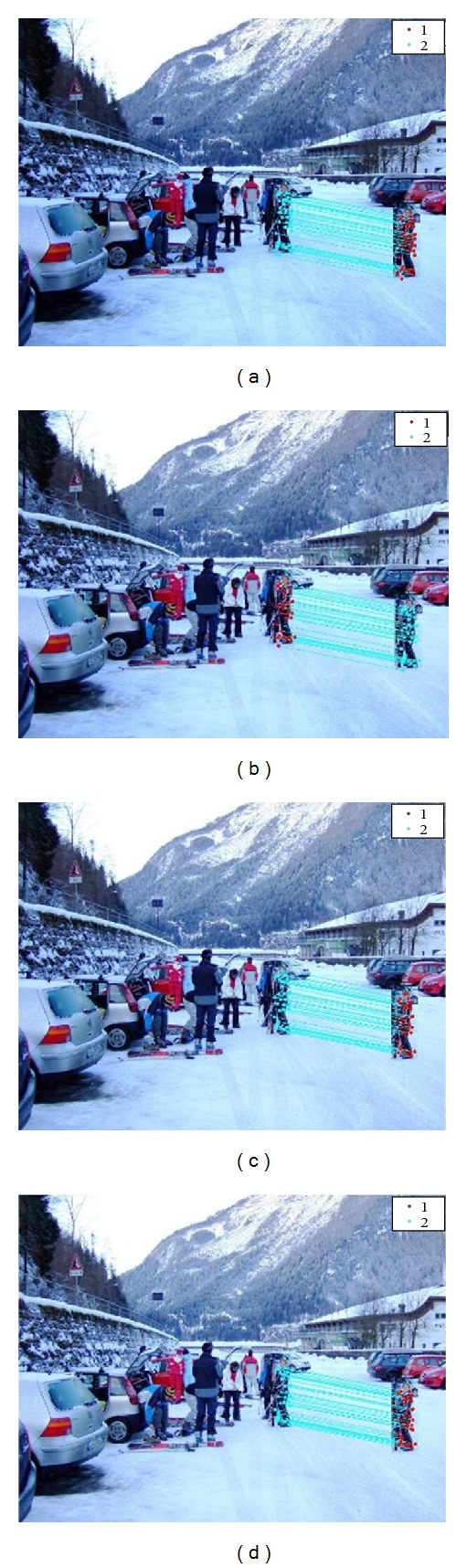
Copy paste detection results for: (a) gamma value = 1.2, (b) gamma value = 1.4, (c) gamma value = 1.6, and (d) gamma value = 1.8.

For checking the robustness of this method we applied different attacks on images.  Figure 5 depicts the detection result in the presence of compression, for JPEG quality factor 20, 40, 60, and 80, respectively.  Figure 6 denotes the detection results in addition of white Gaussian noise whose SNR values are 20, 30, 40, and 50, respectively. In Figure 7, detection results are shown in the presence of Gaussian blurring. The value of the window size is 5 × 5, 7 × 7, and the value of *σ* is taken as 0.5, 1.  Figure 8 represents the detection results in the presence of Gamma correction values 1.2, 1.4, 1.6, and 1.8. In all these images, copied and pasted regions are represented separately by clusters. A line drawn between two key points indicates that this point matches with each other. The performance of detection method is measured in terms of true positive rate (TPR), false positive rate (FPR), and time complexity where

(11) $$\begin{array}{c} \text{TPR}=\frac{\text{images detected as forged being forged}\;}{\text{total number of forged images}}, \end{array}$$

(12) $$\begin{array}{c} \text{FPR}=\frac{\text{images detected as forged being original }}{\text{total number of original images}}. \end{array}$$

TPR is the percentage of forged images, which are correctly identified. FPR is the percentage of the original image which is wrongly identified as a tampered. 

**Figure 9:**
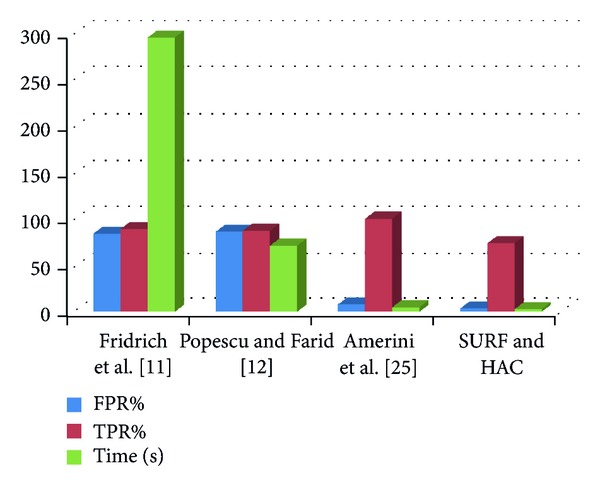
Performance of the methods represented in graph.

The values of FPR, TPR, and time (in seconds) for SURF and HAC methods will be computed. Then, a comparison is performed with other methods. The starting three rows shown in Table 1 are taken from [25] as a benchmark and the fourth row represents the value obtained from SURF and HAC based methods.  Figure 9 represents the result in graphical form. The graph indicates that this method reduces the FPR rate as well as the time complexity. FPR value is approximately 4, which is lower than DCT [11] and PCA [12] methods. Also, the time required to detect the forgery is very low compared to [11] and [12]. TPR value is low which shows one drawback of this method.

## 5. Conclusion and Future Work

In this paper, a method was presented for detecting the duplicate region based on SURF and HAC. The integral image used in SURF reduces the time complexity. SURF has less feature descriptor dimensional size. So that matching applied on SURF descriptor is faster and increases the computation speed as well. The Haar wavelets are used for feature descriptors computation from each keypoint, so descriptors are robust to illumination changes. The experimental results show that SURF feature descriptors are invariant towards different combination of scaling and rotation. In the presence of JPEG compression, Gaussian noise addition and gamma correction attack; this method gives good result. HAC is used here for creating the regions from matched keypoints. HAC is easy to implement and create regions in less time, but satisfactory result is not obtained in terms of the true positive rate. So in the future we would like to replace clustering with suitable image segmentation technique, also we want to utilize this method for multiple duplication detection in a single image. 

## Figures and Tables

**Table 1:** TPR, FPR values (%) and processing time (average per image) for each method.

Methods	FPR%	TPR%	Time (s)
Fridrich et al. [11]	84	89	294.69
Popescu and Farid [12]	86	87	70.97
Amerini et al. [25]	8	100	4.94
SURF and HAC	3.64	73.64	2.85
